# Genomic evidence for evolutionary history and local adaptation of two endemic apricots: *Prunus hongpingensis* and *P. zhengheensis*

**DOI:** 10.1093/hr/uhad215

**Published:** 2023-10-27

**Authors:** Xiaokang Dai, Songzhu Xiang, Yulin Zhang, Siting Yang, Qianqian Hu, Zhihao Wu, Tingting Zhou, Jingsong Xiang, Gongyou Chen, Xiaohua Tan, Jing Wang, Jihua Ding

**Affiliations:** National Key Laboratory for Germplasm Innovation & Utilization of Horticultural Crops, College of Horticulture and Forestry Sciences, Hubei Hongshan Laboratory, Hubei Engineering Technology Research Center for Forestry Information, Huazhong Agricultural University, 430070, Wuhan, Hubei, China; Shennongjia Academy of Forestry, 442499, Shennongjia Forestry District, Hubei, China; Key Laboratory for Bio-Resources and Eco-Environment of Ministry of Education, College of Life Sciences, Sichuan University, 610065, Chengdu, Sichuan, China; Shennongjia Academy of Forestry, 442499, Shennongjia Forestry District, Hubei, China; Shennongjia Academy of Forestry, 442499, Shennongjia Forestry District, Hubei, China; National Key Laboratory for Germplasm Innovation & Utilization of Horticultural Crops, College of Horticulture and Forestry Sciences, Hubei Hongshan Laboratory, Hubei Engineering Technology Research Center for Forestry Information, Huazhong Agricultural University, 430070, Wuhan, Hubei, China; National Key Laboratory for Germplasm Innovation & Utilization of Horticultural Crops, College of Horticulture and Forestry Sciences, Hubei Hongshan Laboratory, Hubei Engineering Technology Research Center for Forestry Information, Huazhong Agricultural University, 430070, Wuhan, Hubei, China; Shennongjia Academy of Forestry, 442499, Shennongjia Forestry District, Hubei, China; Shennongjia Academy of Forestry, 442499, Shennongjia Forestry District, Hubei, China; Shennongjia Academy of Forestry, 442499, Shennongjia Forestry District, Hubei, China; Key Laboratory for Bio-Resources and Eco-Environment of Ministry of Education, College of Life Sciences, Sichuan University, 610065, Chengdu, Sichuan, China; National Key Laboratory for Germplasm Innovation & Utilization of Horticultural Crops, College of Horticulture and Forestry Sciences, Hubei Hongshan Laboratory, Hubei Engineering Technology Research Center for Forestry Information, Huazhong Agricultural University, 430070, Wuhan, Hubei, China

## Abstract

Apricot, belonging to the *Armeniaca* section of Rosaceae, is one of the economically important crop fruits that has been extensively cultivated. The natural wild apricots offer valuable genetic resources for crop improvement. However, some of them are endemic, with small populations, and are even at risk of extinction. In this study we unveil chromosome-level genome assemblies for two southern China endemic apricots, *Prunus hongpingensis* (PHP) and *P. zhengheensis* (PZH). We also characterize their evolutionary history and the genomic basis of their local adaptation using whole-genome resequencing data. Our findings reveal that PHP and PZH are closely related to *Prunus armeniaca* and form a distinct lineage. Both species experienced a decline in effective population size following the Last Glacial Maximum (LGM), which likely contributed to their current small population sizes. Despite the observed decrease in genetic diversity and heterozygosity, we do not observe an increased accumulation of deleterious mutations in these two endemic apricots. This is likely due to the combined effects of a low inbreeding coefficient and strong purifying selection. Furthermore, we identify a set of genes that have undergone positive selection and are associated with local environmental adaptation in PHP and PZH, respectively. These candidate genes can serve as valuable genetic resources for targeted breeding and improvement of cultivated apricots. Overall, our study not only enriches our comprehension of the evolutionary history of apricot species but also offers crucial insights for the conservation and future breeding of other endemic species amidst rapid climate changes.

## Introduction

Apricot, a member of the *Armeniaca* section of Rosaceae, is one of the economically important fruit trees that have been cultivated worldwide and in temperate regions [1]. There is a rich diversity of apricot germplasm, with at least 10 apricot species originating in China, including two well-known species, *Prunus mume* Sieb. (mei) and *Prunus armeniaca* L. (common apricot) [2]. The apricot offers valuable genetic resources for apricot breeding and improvement. However, some local and/or natural wild species of apricot are ignored, abandoned, and even at risk of extinction. Of these, *P. hongpingensis* Yu & Li (PHP) and *P. zhengheensis* Zhang & Lu (PZH) are two southern China endemic apricots with a very narrow distribution. PHP is found only in the mountains of Shennongjia Forestry District, Hubei Province, China [3], while PZH has a restricted distribution in Zhenghe County, Fujian Province, China [4]. Genomic studies of common apricot, mei and other related species have shed light on the evolutionary and domestication history of apricots [5–10], yet have seldom involved research on these two apricots. Moreover, a substantial debate persists regarding the classification status of these two endemic apricot species, primarily stemming from discrepancies between morphological characteristics and molecular data [3, 4, 11]. As a result, the phylogeny and evolutionary history of these two endemic apricots remain unclear.

Given their restricted geographic range and small population sizes, rare and endemic species face heightened susceptibility to genetic diversity depletion and reduced fitness arising from the effects of genetic drift and inbreeding [12]. This makes them more vulnerable to extinction, particularly in the face of accelerated climate changes and increased human activities [13–16]. In addition, the accumulation of deleterious mutations associated with the small population size may further cause a reduction in fitness and increase the extinction risk for these endemic species [13]. A previous study has reported that Central Asian *P. armeniaca* experienced bottlenecks during the Pleistocene, followed by rapid demographic expansion [5]. Multiple gene flow events have been detected among apricot populations in different geographic areas [5, 8, 17], which may counterbalance the influence of bottleneck effects. However, the geographically restricted distribution of PHP and PZH may limit their gene flow with other nearby species. This could lead to continuously reduced genetic diversity following the bottleneck events and an increased risk of extinction. Thus, it is essential to understand the patterns of genetic diversity, interspecific gene flow events, and changes in effective population size in order to protect them. This is especially important considering the multifactorial pressures, such as anthropic activities and rapid climate changes, that their geographically restrictive ecosystem and regions may face. Furthermore, most cultivated apricots, derived from *P. armeniaca*, are primarily cultivated in northern China [5] and are not well suited for cultivation in the hot and rainy weather of southern China. Therefore, the exploration of genes and variants associated with adaptation to the local environments in PZH, which thrives in southern China, can greatly benefit the domestication and future breeding of apricots in this region. Moreover, the high-altitude wild PHP apricot, which is only found in the mountains of Shennongjia Forestry District (~1800–1900 m), represents a valuable germplasm resource for high-altitude apricot domestication.

In this study, we first assembled the high-quality chromosomal-scale genome for these two endemic apricots (PHP and PZH) and then conducted a comprehensive analysis to characterize their phylogenetic relationship, genetic diversity, demographic history, and degree of hybridization with common wild and cultivated apricots, and explored the genomic basis of their local climate adaptation. A large-scale single nucleotide polymorphisms (SNPs) data set from 72 high-coverage genomes of five apricot species was used, including 19 newly resequenced accessions in this study (Supplementary Data Table S1; 13 and 6 individuals of PHP and PZH, respectively) and 53 accessions obtained from previous studies (Supplementary Data Table S2; 10 wild *P. armeniaca*, 12 wild *Prunus sibirica* and 31 cultivated *P. mume*) [5, 18]. Utilizing this genomic data, our objective was to investigate the following questions. (i) What are the phylogenetic relationships of PHP, PZH, and other species in the *Armeniaca* section? (ii) How did species divergence occur and what are the demographic histories of the species? (iii) Did ancient interspecific gene flow occur between these two endemic species and other apricot species? (iv) Are there some genomic regions that show strong positive selection for local environments in PHP and PZH?

## Results

### Morphological characters of PHP and PZH

**Figure 1:**
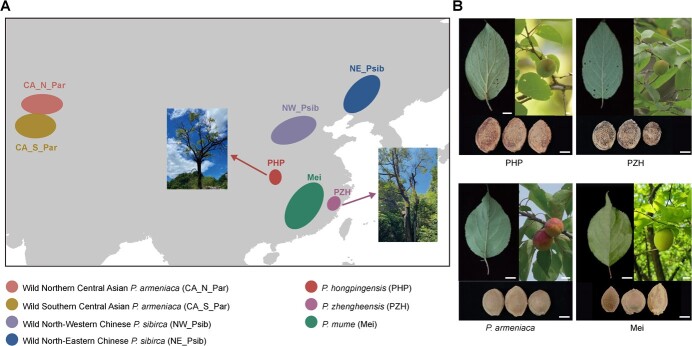
Distribution and morphological characters of *Armeniaca* section species. **A** Geographic distribution of apricot species used in this study. Different color ranges in the map indicate the distribution of individuals in each population. **B** Photographs of leaves, fruit, and fruit stone of PHP, PZH, *P. armeniaca*, and mei. Scale bar = 1 cm.

Both PHP and PZH are found in the wild forests of southern China and have a very narrow distribution (Fig. 1A). When comparing the morphological characteristics of PHP/PZH with other described *Prunus* species [11], we observed two obvious morphological differences. Firstly, the leaves of *P. armeniaca* and *P. mume* are oval-shaped with an acuminate apex and are glabrous on both surfaces. In contrast, leaves were elliptic to elliptic–ovate with densely pubescent in the vein axils for both PHP (yellowish brown villous abaxially) and PZH (grayish villous abaxially) (Fig. 1B). Secondly, the endocarps of *P. armeniaca* and *P. mume* are typically smooth, with rare grooves or rugoses. However, the endocarps of both PHP and PZH are ellipsoid in shape, scabrous, shallowly reticulate, and pitted (Fig. 1B). The shared morphological characteristics suggested a closer relationship between PHP and PZH.

### Genome sequencing, assembly, and annotation of PHP and PZH

**Figure 2:**
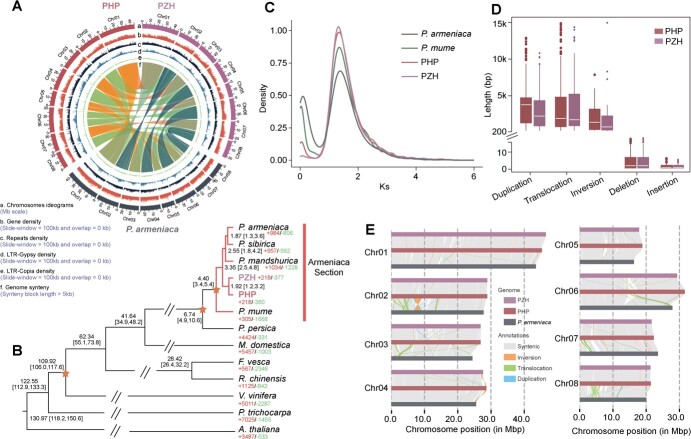
Genomic features and evolution of PHP and PZH. **A** Circos display of genomic features of PHP and PZH. The Circos tracks represent (a) chromosome ideogram (Mb scale), (b) gene density (slide window = 100 kb, overlap = 0 kb), (c) repeat density (slide window = 100 kb, overlap = 0 kb), (d) LTR-Gypsy density (slide window = 100 kb, overlap = 0 kb), (e) LTR-Copia density (slide window = 100 kb, overlap = 0 kb), (f) genome synteny among PHP, PZH, and *P. armeniaca* (synteny block length >5 kb). **B** Divergence time tree estimated using the MCMCtree of the PAML package. Numbers on the nodes are median age estimates and 95% highest posterior densities (Mya). Fossil calibration points are indicated by orange stars. Expansion and contraction gene families are presented under each species. **C** Synonymous substitution rate (*K*_s_) density distributions of intraspecies synteny of PHP, PZH, *P. armeniaca*, and mei. **D** Length of SVs detected between the genomes of PHP and *P. armeniaca* and the genomes of PZH and *P. armeniaca*. **E** Structural variation between the genomes of PHP and *P. armeniaca* and the genomes of PHP and *P. armeniaca*, including synteny, inversions, translocations, and duplications.

To obtain high-quality genomic sequences, we performed chromosome-level assembly of PHP and PZH genomes. Using PacBio HiFi long-read sequencing technology, we generated a total of ~10.21 and ~ 12.25 Gb of clean sequence data for PHP and PZH, respectively, representing ~50-fold coverage of the whole genome for both species. To achieve a higher-quality chromosome-scale assembly, we also generated 100-fold coverage (~15 Gb) high-throughput chromatin conformation capture (Hi-C) data for PHP (Supplementary Data Fig. S1). For the assembly of PZH, we used RagTag software [19] for automating assembly scaffolding (Supplementary Data Fig. S2). The final chromosome-level genome assembly captured 213.47 Mb (PHP) and 209.82 Mb (PZH) of the genome sequence, with 17.86 Mb (PHP) and 24.17 Mb (PZH) contig N50 length and eight pseudochromosomes for each species (Table 1, Fig. 2A). BUSCO (Benchmarking Universal Single-Copy Orthologs) analyses indicated that 98.1 and 98.7% of the predicted genes were full length for PHP and PZH, indicating that the genome assemblies of both species were highly complete. To annotate the genes in our new genome assemblies, we employed a combination of the transcriptome-, homology-, and *ab initio*-based approaches. Finally, we identified 30 416 genes and 33 624 proteins in PHP, 32 534 genes and 35 516 proteins in PZH, with an average coding sequence length of 1106 and 1060 bp for PHP and PZH, respectively (Table 1, Fig. 2A). The genomic data of PHP and PZH provide valuable genetic resources for prospective studies in functional genomics and targeted breeding initiatives within the apricot. The genomic data of PHP and PZH offer significant genetic reservoirs for prospective studies in functional genomics and targeted breeding initiatives within the apricot genus.

### Genome evolution and structure comparative analysis

Using OrthoFinder, we assigned 492 822 genes (92.5% of the total genes across the 12 species genomes) to 38 965 orthogroups. Among these orthogroups, 228 746 genes clustering in 9093 groups were conserved in all examined plants. Gene-family evolution analysis showed that there were 218 expanded families and 380 contracted families in PHP, while PZH had 218 expanded families and 377 contracted families Moreover, a compilation of 1198 orthogroups, in which each orthogroup contained a minimum of 84.6% of the species featuring single-copy genes, was utilized to construct the phylogenetic tree and the species divergence time was determined through molecular dating (Fig. 2B). Our results showed that PHP and PZH clustered together and were split from other species of the *Armeniaca* section. The estimated time of the split between PHP and PZH was ~1.92 million years ago (Mya) [95% highest posterior density (HPD) 1.2–3.2 Mya; early Pleistocene]. In addition, mei is located at the base position of the *Armeniaca* section, consistent with a previous study [5]. The *K*_s_ density map showed a shared peak (*K*_s_ value ~1.3) in all four species, which reflects the γ duplication event that occurred in the early evolutionary history of eudicots (Fig. 2C). To determine structural variations (SVs), we then compared the new assembly genome of PHP and PZH with the reference *P. armeniaca* using the whole-genome comparison tool SyRI [20]. In total, we identified 262 457/266 213 SVs comprising 166 396/167 973 insertions, 95 179/97 597 deletions, 441/362 inversions, 201/144 translocations, and 240/137 duplications in these two comparisons (PHP versus *P. armeniaca* and PZH versus *P. armeniaca*). Large-fragment SVs (>200 kb) were found in the inversions, translocations, and duplications (Fig. 2D). Notably, the longest inversion was found in chromosome 2 for PHP (2.25 Mb in Chr2:6 752 002–9 113 188) and PZH (2.41 Mb in Chr2:6 463 938–8 988 934), harboring 209 and 243 protein-coding genes in PHP and PZH, respectively (Fig. 2E, Supplementary Data Tables S3 and S4).

**Table 1:** Genomic assembly statistics for PHP and PZH.

Assembly	PHP	PZH
Final assembly length (Mb)	213.47	209.82
Scaffold N50 (L50) (Mb)	28.66	26.87
Maximum length of scaffold (Mb)	45.29	46.48
Number of contigs	732	392
Contig N50 (L50) (Mb)	17.86	24.17
Number of genes	30 416	32 534
Number of predicted proteins	33 624	35 516
Mean coding sequence length (bp)	1106	1060
BUSCO score (%)	98.10	98.70

### Genetic divergence of PHP and PZH

To investigate the population structure and nucleotide diversity of PHP and PZH, we generated whole-genome resequencing data for 13 and 6 individuals of PHP and PZH, respectively (Supplementary Data Table S1). We also studied genome resequencing data of 10 wild *P. armeniaca*, 12 wild *P. sibirica*, and 31 cultivated *P. mume* that were previously published (Supplementary Data Table S2) [5, 6]. A total of 72 of the *Armeniaca* accessions were used for variant calling. After randomly selecting two samples in each species/population and using the clean reads for read mapping based on the reference genomes of newly assembled PHP and PZH, as well as *P. armeniaca* to perform variant calling, we detected similar numbers of SNPs based on each of the three reference genomes (Supplementary Data Fig. S3A). The genetic clustering based on each of the three SNP datasets also showed highly consistent results from principal component analysis (PCA) (Supplementary Data Fig. S3B). However, considering that more samples belong to the *P. armeniaca* clade in this study, we finally chose the latest high-quality apricot genome of *P. armeniaca* as the reference to perform variant calling across the 72 accessions. Approximately 300 Gb of high-quality reads were aligned against the reference genome of apricot [5]. After SNP calling, a set of 18 187 408 SNPs was retained and used for the following population-based genomic analyses.

**Figure 3:**
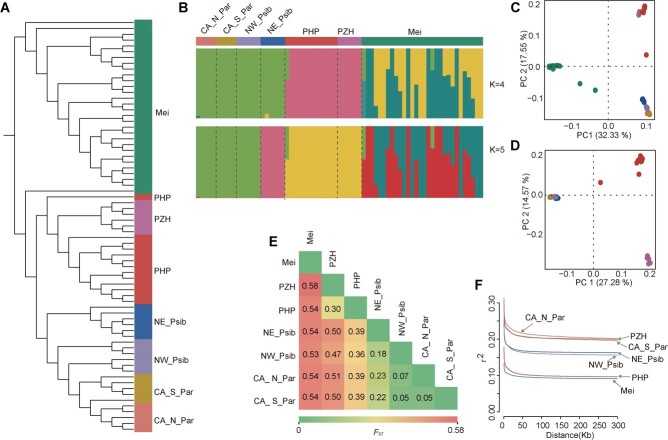
Population delimitation of five apricot species. **A** A maximum likelihood phylogenetic tree was constructed by IQ-TREE with an ascertainment bias correction (+ASC) model and visualized by FigTree. All the bootstrap values are 100 along the branches. **B** Population structure by ADMIXTURE analysis for the optimal *K* = 4 and 5. Each color-coded bar corresponds to an individual, and the segmented colors illustrate the proportions of ancestral components. **C** PCA based on genomic SNPs of all accessions. **D** PCA constructed after the removal of all *P. mume* individuals. The colors in (**C**) and (**D**) correspond to those in (**A**). **E** Pairwise population differentiation was estimated using *F*_ST_. **F** Decay of LD was calculated based on *r*^2^ of all apricot accessions.

Using these high-quality SNPs, we conducted a phylogenetic analysis to explore the relationships among 72 *Armeniaca* accessions. The result showed that all individuals of PHP and PZH clustered with *P. sibirica* (NE_Psib and NW_Psib) and *P. armeniaca* (CA_N_Par and CA_S_Par), while all accessions of mei were grouped together at the base position (Fig. 3A). Population structure analysis also supported the differentiation of PHP and PZH from other populations (Fig. 3B, Supplementary Data Fig. S4). Interestingly, one admixed individual of PHP was identified, which exhibited a genetic background from CA_N_Par, CA_S_Par, and NW_Psib (Fig. 3B). Consistently, the PCA analysis results showed that most PHP (except one individual) and all PZH individuals formed a single cluster that was separate from other species (Fig. 3C). When excluding mei, PHP and PZH displayed in a different quadrant, and both were separate from *P. sibirica* (NE_Psib and NW_Psib) and *P. armeniaca* (CA_N_Par and CA_S_Par) (Fig. 3D). The *F*_ST_ results suggested that there was a strong differentiation between PHP/PZH and other apricot populations (Fig. 3E), with PHP being closer to *P. armeniaca* and *P. sibirica* than PZH. In addition, consistent with the phylogenetic and structure analysis results, a low *F*_ST_ value was observed among the CA_N_Par, CA_S_Par, and NW_Psib populations, indicating a close relationship among them.

We estimated the decay of linkage disequilibrium (LD) based on average *r*^2^ for SNPs within 300 kb from each other. PHP and *P. mume* displayed the fastest decay of LD, while PZH and *P. armeniaca* (CA_N_Par and CA_S_Par) displayed the slowest decay rate (Fig. 3F). The genome-wide levels of genetic diversity (*π*) were also calculated. The level of *π* was not significantly different across each of the groups, with the lowest *π* (7.11e−3) in PZH. Furthermore, both PHP and PZH showed higher Tajima’s *D* values compared with other populations (Supplementary Data Table S5).

### Divergence and demographic history

**Figure 4:**
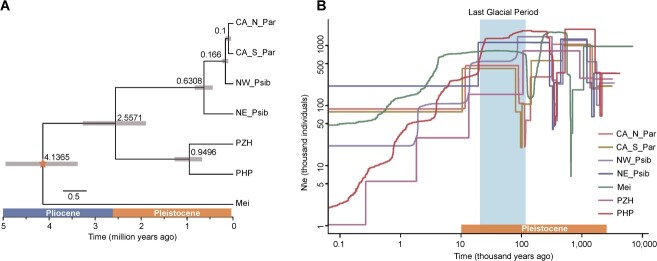
Divergence and demographic histories of the different apricot populations. **A** Population divergence times based on a SNAPP analysis. The estimated divergence times at specific nodes were guided by a singular calibration, denoted by an star. Median age estimates, accompanied by 95% HPDs (Mya), have been provided for each respective node. **B** Comparison of historical effective population size (*N*_e_) changes across the various populations.

The time tree revealed that the split between the PHP/PZH clade and two other apricots (*P. armeniaca* and *P. sibirica*) occurred ~2.55 Mya (early Pleistocene) with a 95% HPD interval of 1.89–3.26 Mya (Fig. 4A). The estimated age of the split between PHP and PZH was around 0.94 Mya (95% HPD 0.67–1.26 Mya; middle Pleistocene). The demographic history of each population was inferred based on the unfolded SFS using Stairway Plot 2 [21]. The results showed that PHP underwent a sharp demographic bottleneck around 0.52–1.64 Mya which coincided with NE_Psib and NW_Psib (Fig. 4B). Meanwhile, the bottleneck of the two populations of *P. armeniaca* (CA_N_Par and CA_S_Par) occurred in the Last Glacial Maximum (26 500–19 000 years ago), when the temperature underwent an extreme decline [22]. After the bottleneck, a rapid demographic expansion was observed in PHP, NE_Psib, NW_Psib, CA_N_Par, and CA_S_Par. However, compared with the other populations, the effective population size of PZH showed a step-down pattern without a significant bottleneck. Notably, the effective population sizes (*N*_e_) of PHP and PZH have declined extremely in recent years, while the *N*_e_ of other populations has remained relatively stable.

### Heterozygosity, inbreeding level, and deleterious variants

**Figure 5:**
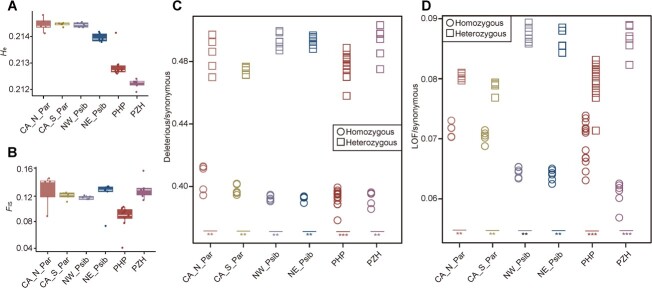
Assessment of heterozygosity, inbreeding level, and deleterious variants. **A** Expected heterozygosity (*H*_e_) and inbreeding coefficient (*F*_IS_) of all populations, excluding mei. **C**, **D** Ratio of derived deleterious (**C**) and LOF (**D**) variants to synonymous variants in homozygous (circle) and heterozygous (square) tracts per individual. The ratio differences between homozygous and heterozygous tracts were calculated using the Wilcoxon rank-sum test (**P < .01; ***P < .001).

As PHP and PZH are both rare and endemic, we examined their heterozygosity and inbreeding levels, and the accumulation of deleterious variants across sampled individuals. The result showed lower expected heterozygosity (*H*_e_) in PHP and PZH, indicating limited genetic variability in these two endemic species (Fig. 5A). Previous studies suggested that high inbreeding coefficients may cause loss of heterozygosity and result in reduced fitness [23, 24]. However, we did not find an increase in inbreeding coefficient (*F*_IS_) in PHP and PZH, and the lowest level of genomic inbreeding was observed in PHP (Fig. 5B). We detected the distributions of three types of variation in protein-coding genes using SIFT 4G software to assess the deleterious mutations [25]. We observed a higher ratio of heterozygous-derived deleterious and LOF (loss of function) mutations to synonymous mutations in PHP and PZH (Fig. 5C and D). This suggests that the deleterious and LOF mutations are more likely to act recessively and be maintained in a heterozygous state in these species (Fig. 5C and D). Compared with other species, we did not observe an increase in the ratios of derived functional variants (both deleterious and LOF variants) relative to synonymous variantsin PHP and PZH (Fig. 5C and D). These results suggest that the efficient purging of strongly deleterious alleles may enhance the ability to avert extinction during the evolution of PHP and PZH due to their long-term small population sizes.

### Whole-genome introgression analysis

**Figure 6:**
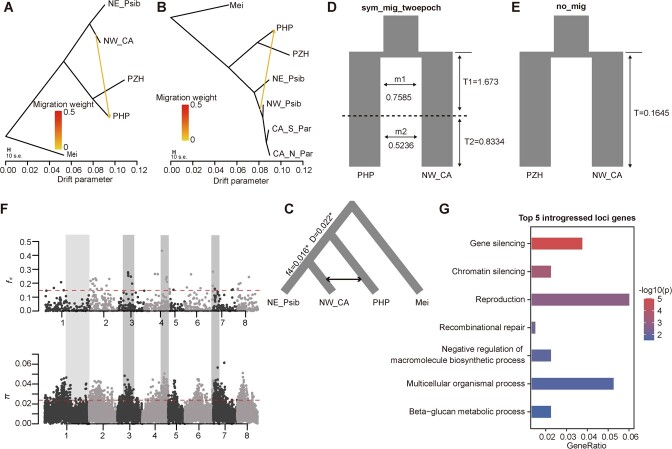
Gene flow analysis using various methods. **A** TreeMix topologies with one suggested gene flow event between the Mei, NE_Psib, NW_CA clade, PZH, and PHP populations. **B** Gene flow identification between PHP and CA_NW clade. We divided the CA_NW clade into CA_N_Par, CA_S_Par, and NW_Psib and then performed TreeMix analysis with one suggested gene flow event (orange arrow). **C** The suggested gene flow event in (**A**) was further evaluated using a four-taxon test. **D**, **E** Population genetic model comparison results between population sets PHP and NW_CA (**D**), and PZH and NW_CA (**E**). **F** Distribution of modified *f*_d_ statistics (PHP and NW_CA) and genetic diversity of selective sweeps in the 100-kb non-overlapping window. The red dashed lines indicate the threshold (5%) of modified *f*_d_ statistics and *π*. The light gray range indicates the low *π* and *f*_d_ values, and dark gray indicates the high *π* and *f*_d_ values. **G** GO enrichment analysis of 756 genes with the modified *f*_d_ value higher than the top 5%.

In this study, the potential interspecific gene flows were examined using TreeMix, ABBA-BABA test, dadi_pipeline, and modified *f*_d_ statistics. Through phylogenetic, structure, and *F*_ST_ analysis, a close relationship was found among CA_N_Par, CA_S_Par, and NW_Psib. Previous studies have also indicated that the North-Western *P. sibirica* (NW_Psib in this study) should be assigned to *P. armeniaca* [5]. Therefore, we defined CA_N_Par, CA_S_Par, and NW_Psib as CA_NW clade and subsequently performed whole-genome introgression analysis between PHP/PZH and this clade, as well as other apricots. TreeMix analysis identified a significant gene flow from the CA_NW clade to PHP (Fig. 6A). To further investigate the gene flow between PHP and the CA_NW clade, we divided the CA_NW clade into CA_N_Par, CA_S_Par, and NW_Psib and then performed TreeMix analysis. The result still showed a significant gene flow from the CA_NW clade (including CA_N_Par, CA_S_Par, and NW_Psib) to PHP (Fig. 6B). The ABBA-BABA tests also indicated a significant gene flow event (*f*_4_ = 0.015, *D* = 0.022) between the CA_NW clade and PHP (Fig. 6C). Moreover, dadi was used to scan and compare the likelihoods of seven different introgression models (Supplementary Data Fig. S5). For PHP, the ‘sym_mig_twoepoch’ model displayed the highest log-likelihood score and the lowest Akaike information criterion (AIC), suggesting two symmetric gene flow events during the divergence and secondary contact (Fig. 6D). On the other hand, the ‘no_mig’ model was the best-fitting model for PZH, indicating divergence without migration (Fig. 6E).

To identify the genomic regions that were likely shared by introgression between PHP and the CA_NW clade, we estimated the excess of shared derived mutations using the modified *f*_d_ statistic (Fig. 6F). A total of 756 genes were identified from 42 windows with modified *f*_d_ values in the top 5% (Supplementary Data Table S6). The introgression regions with high *f*_d_ values were also observed to show higher genetic diversity compared with other regions (Fig. 6D). GO enrichment analysis revealed that the top 5% of genes were involved in basic functions, such as ‘reproduction’ and ‘multicellular organismal process’ (Fig. 6G, Supplementary Data Table S7).

### Identification of selective sweeps in PHP and PZH

**Figure 7:**
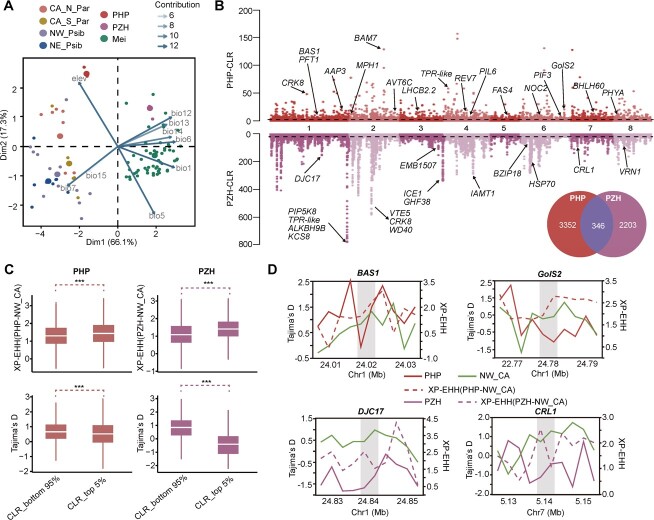
Selection signatures in the genomes of PHP and PZH under local adaptation. **A** PCA for environmental variables of five apricot species (seven populations). The percentages of variation explained by each principal component are indicated in parentheses. Elev, elevation; bio1, annual mean temperature; bio5, maximum temperature of warmest month; bio6, minimum temperature of coldest month; bio7, annual temperature range (bio5 − bio6); bio12, annual precipitation; bio13, precipitation of wettest month; bio14, precipitation of driest month; bio15, precipitation seasonality (coefficient of variation). **B** Manhattan plot of CLR of PHP and PZH. The dashed line indicates the CLR value threshold (top 5%). Functionally characterized candidate genes are denoted (Supplementary Data Tables S12 and S13). Venn diagram of the top 5% genes of PHP and PZH. **C** Tajima’s *D* statistic and XP-EHH test for the top 5% and bottom 95% genes in CLR analysis. Asterisks indicate the degree of significance in both the Wilcoxon rank-sum test and the *t*-test (***P < 0.001). **D** Signals of artificial selection in *BAS1* and *GolS2* genes in PHP and *DJC1* and *CRL1* genes in PZH.

The distribution of PHP was restricted to altitudes of around 2000 m in the mountains of Shennongjia Forestry District, Hubei Province, China [3]. PZH, on the other hand, was discovered in Fujian Province, China, which is characterized by high humidity and hot weather [4]. The PCA in ecological space revealed that these two species showed different environmental characteristics from the wild apricots that are currently used for domestication (Fig. 7A). Altitude was identified as the primary factor separating PHP from other species/populations, while the precipitation showed a high association with PZH (Fig. 7A). Overall, the two species are likely subjected to different bio- and/or abiotic selective pressures due to their distinct environmental distributions.

To examine the footprint of selection, we scanned the genomes for PHP and PZH using the composite likelihood ratio (CLR) test implemented in SweeD. We identified selective sweep signatures in 3698 and 2549 genes for PHP and PZH, respectively (Supplementary Data Tables S8 and S9). Most of the selected genes were unique to either PHP or PZH (Fig. 7B). The selected genes of PHP were highly enriched in the GO terms and KEGG pathways related to alpine adaptation, such as ‘response to stress’, ‘flavonoid biosynthesis’, ‘photosynthesis–antenna proteins’, ‘DNA repair’, and ‘response to light stimulus’ (Supplementary Data Fig. S6 and Supplementary Data Table S10). Among them, several genes related to light and stress responses were identified, including *PHYB ACTIVATION-TAGGED SUPPRESSOR1* (*BAS1*), *PHOTOSYSTEM II LIGHT-HARVESTING COMPLEXES GENE 2.2* (*LHCB2.2*), *PHYTOCHROME INTERACTING FACTOR 3-LIKE 6* (*PIL6*), *PHYTOCHROME INTERACTING FACTOR 3* (*PIF3*), *GALACTINOL SYNTHASE 2* (*GolS2*), and *PHYTOCHROME A* (*PHYA*) (Fig. 7B). In contrast, the selected genes of PZH were mainly involved in environmental adaptation signals and metabolism pathways, such as ‘cell wall modification’, ‘cell division’, ‘vacuolar transport’, ‘photosynthesis’, ‘cutin, suberin and wax biosynthesis’ (Supplementary Data Fig. S7 and Supplementary Data Table S11). Many of these selected genes were found to be associated with the humidity and hot weather, including *DNA J PROTEIN C17* (*DJC17*), *VITAMIN E PATHWAY GENE5* (*VTE5*), *GLYCOSYL HYDROLASE FAMILY 38 PROTEIN* (*GHF38*), *BASIC LEUCINE-ZIPPER 18* (*BZIP18*), *PUTATIVE EMBRYO DEFECTIVE 1507* (*EMB1507*), and *CINNAMOYL COA: NADP OXIDOREDUCTASE-LIKE 1* (*CRL1*) (Fig. 7B).

The selected genes were further verified by Tajima’s *D* statistic and the XP-EHH (cross-population extended haplotype homozygosity) test. The results showed that the top 5% of genes with selection signatures exhibited significantly lower Tajima’s *D* values and higher XP-EHH values in both PHP and PZH (Fig. 7C). For instance, we found two light response genes, *BAS1* and *GolS2*, which were additionally supported by a high XP-EHH and a significantly low Tajima’s *D* statistic in PHP compared with the CA_NW clade (Fig. 7D). *BAS1* is involved in brassinosteroid (BR) catabolism in *Arabidopsis* and plays important roles in cotyledon expansion, hypocotyl elongation repression, and flowering time regulation [26, 27]. *GolS2* is known to be a key gene in response to various environmental stresses [28–30]. Additionally, the *CRL1* gene, which is associated with submergence stress and increased tolerance to submergence [31], exhibited significantly low Tajima’s *D* values in PZH and a high XP-EHH value (Fig. 7D). Taking these results together, the two endemic apricots contain unique selected genes with important biological functions related to their local environmental adaptation.

## Discussion

Apricot is an economically important crop fruit that has been domesticated worldwide [5]. Most cultivated apricots originated from *P. armeniaca* [5, 10]. However, the wildly endemic apricots, such as PHP and PZH, contain unique genetic resources that can be used for targeted breeding and cultivar improvement. In this study, we successfully assembled the chromosome-level high-quality genome of PHP and PZH by integrating PacBio HiFi long-read sequencing and Hi-C scaffolding technologies (Table 1, Fig. 2A). These reference genomes will greatly facilitate further exploration of their unique and valuable genetic compositions, which can be used for future breeding and cultivar improvement in apricots.

### Climate change during the Pleistocene likely induced speciation of PHP and PZH

Referring to the evolutionary position of PZH, previous studies based on the chloroplast genome suggested PZH was more closely related to *P. mume* [4], while PHP was more related to *P. armeniaca* [3]. However, the whole-genome sequencing analysis in this study revealed that both PHP and PZH are grouped with *P. armeniaca* (Figs 2B, 3A, and 4A). The conflict between the plastid phylogeny and our results from the genomic nuclear data may be due to the process of chloroplast capture events [32, 33]. Chloroplast capture events frequently happened in species with sympatric distribution and reproductive compatibility [34]. Compared with PHP, PZH shows higher *F*_ST_ values with the populations of *P. armeniaca* and *P. sibirica*, indicating stronger population differentiation. Furthermore, our results show a sister phylogenetic relationship between PHP and PZH*,* which has never been mentioned in previous studies. This result was confirmed by their similar morphological characters (Fig. 1B). The divergence between PHP and PZH was estimated to have likely occurred during the Pleistocene (Fig. 4A). The glacial–interglacial cycles during the Pleistocene, frequently fragmenting the geographical distributions of populations, have been reported to play an important role in driving the processes of diversification and speciation [35]. Although southern China was probably not glaciated during this period [36], the cyclical cooling–warming climate during the Pleistocene may still induce many species to experience periodic habitat expansions and contractions [37]. Therefore, we hypothesize that the expansion and contraction of the species induced by climate change during the middle Pleistocene may have caused geographical isolation between PHP and PZH. The habitat preference in their long-term local environmental adaptation would further enhance their isolation and prevent further gene flow, which would eventually lead to allopatric speciation.

### Avoiding extinction by purging deleterious mutations despite declining *N*_e_ in recent years

The ancient bottlenecks experienced by the populations of PHP, PZH, and other species were expected to substantially reduce genetic diversity. However, extensive introgressions can counterbalance the effects of reduced genetic diversity, enhance adaptation to diverse environments, and aid demographic expansion [38]. Interspecific introgressions have been frequently observed in various plant species [5, 7, 10, 17, 18]. In our study, ancient bottlenecks were detected in all populations followed by a rapid demographic expansion, except for PZH (Fig. 4B). Subsequently, the *N*_e_ of all the populations slightly decreased after the LGM (Fig. 4B), which was consistent with the paleovegetation data that revealed considerable reduction of the Chinese temperate forests during this period [39, 40]. However, unlike other species, the decreased population sizes were not alleviated in PHP and PZH until recently. We speculate that the geographically restricted distribution and the limited gene flow with other species may be the primary reasons for this, which was supported by the lowest genetic diversity and highest Tajima’s *D* of PZH among all the species we studied. Nevertheless, the lower genetic diversity was not found in PHP, possibly due to recent gene flow between PHP and the CA_NW clade (Fig. 6).

Notably, both PHP and PZH possessed a relatively low level of heterozygosity in the genome, largely owing to the long-term small population sizes in both species that are likely caused by their past demographic histories. In general, when deleterious mutations are recessive, they are likely to be masked in heterozygous conditions [41]. The decrease in heterozygosity can expose recessive deleterious mutations. We observed much higher rates (deleterious/ synonymous and LOF/ synonymous) at heterozygous sites compared with homozygous sites in the all populations (Fig. 5C and D). However, after assessing the deleterious mutation load by calculating the ratio of deleterious (including LOF) to synonymous variants and comparing PHP and PZH with other species/populations, we did not observe a significantly increased accumulation of deleterious mutations in these two endemic apricots (Fig. 5D). Moreover, we did not find an increased *F*_IS_ in either PHP or PZH (Fig. 5B). Contrary to the expectation that endemic species are more likely to accumulate deleterious mutations due to their restricted and small populations [42], our findings suggest that persistent small population sizes over the long term can constrain the accumulation of highly deleterious recessive mutations through robust purifying selection, thereby averting extinction [13, 43, 44]. Thus, the effective elimination of highly deleterious recessive alleles and the avoidance of inbreeding can elucidate the absence of deleterious mutation accumulation in PHP and PZH.

### Selective signatures of local environmental adaptation

Adaptation to local environments in natural populations commonly manifests as discernible signatures of positive selection across the genome [45, 46]. These signatures are discernible from the extensive SNP data, involving the identification of statistical outliers and the characterization of genes subject to selection. However, previous studies on apricots have mainly focused on the domestication history and identified selected genes related to fruit quality and disease resistance [5, 10]. Limited research has addressed natural selection in the local environmental adaptation of wild endemic apricot species and populations. Our results suggest that PHP and PZH occupy specific ecological niches that are significantly different from those of the previously studied northern apricots (Fig. 7A). Specifically, PHP is distributed in the alpine condition with high altitudes, and PZH is characterized by a heavy precipitation climate.

Using the CLR test, we have identified the genomic regions under strong positive selection in both PHP and PZH. Focusing on the selected signals of PHP, we found a collection of genes involved in the alpine environmental adaptation (Supplementary Data Fig. S6). The extremely intense UV radiation at high altitude will increase the frequency of somatic homologous DNA rearrangements and may cause DNA damage [47]. Genes involved in DNA repair and abiotic stress responses have been shown to be under positive selection in many alpine plants, including Tibetan cultivated apricots [48–51]. In addition, strong UV can affect plant growth and developmental processes such as photomorphogenesis and photosynthesis [52, 53]. We observed strong selection signatures in genes associated with the phytochrome family and its related genes (*PHYA*, *PIF3*, *PIL6*, *PFT1*, *BAS1*, and *LHCB2.2*) (Fig. 7A). Moreover, high-light stress-response genes were also found under selection, such as *GolS2* [54, 55], *MPH1* [56, 57], and *REV7* [58]. Overall, these genes likely play important roles in PHP local adaptation in alpine conditions and can serve as key genetic resources for future targeted breeding. In contrast, PZH is only found in southern China, which is characterized by high humidity and hot weather. PCA based on environmental variables showed that precipitation-related climates (bio12, 13, and 14) strongly separate PZH from the other apricots (Fig. 7A). Among the selected genes in PZH, we identified the *CRL1* gene, which responds to submergence stress and increases tolerance to submergence in the high-precipitation population of *Populus koreana* [31]. We also identify a set of temperature-associated genes, including *DJC17*, *HSP70*, *HSC70*, and *EMB1507*. The results suggest that genes involved in precipitation and temperature-related adaptation are likely to be important for PZH to adapt to the unique environmental conditions in southern China. Overall, the identification of genes under strong natural selection during the adaptation of PZH and PHP greatly extends our understanding and provides valuable insights for further breeding efforts.

## Materials and methods

### Plant materials and DNA sequencing

For PacBio long-read sequencing, leaf samples of PHP and PZH were collected and genomic DNA was isolated using the CTAB method [59]. Subsequent sequencing was performed on the PacBio Sequel II CLR platform (Bioyi Biotechnology Co., Ltd, Wuhan, China). Hi-C libraries were prepared and sequenced on the Illumina Novaseq/MGI-2000 platform (Bioyi Biotechnology Co., Ltd, Wuhan, China) for the chromosome-level genome assembly from the PHP. To facilitate gene annotation, total RNA of PHP was isolated from the roots, leaves (young and mature), stems, and vegetative shoot apices using the CTAB–LiCl method [60] and quantified using the Agilent Bioanalyzer 2100 System. High-quality RNA of various tissues was mixed and used to construct one cDNA library, and subsequent sequencing was performed on the Illumina HiSeq 2500 sequencer (Bioyi Biotechnology Co., Ltd, Wuhan, China) with a paired-end sequencing strategy.

For whole-genome resequencing, leaf samples were collected from 13 PHP and 6 PZH individuals in the field, representing the most endangered individuals discovered to date. The individual geographic coordinates and sequence information are provided in Supplementary Data Table S1. Paired-end libraries were constructed for 150-bp sequencing on an Illumina HiSeq 2500 sequencer (Bioyi Biotechnology Co., Ltd, Wuhan, China). We also included 12 wild *P. sibirica* (NE_Psib and NW_Psib), 10 wild *P. armeniaca* (CA_N_Par and CA_S_Par), and 31 *P. mume* cultivars (mei) reported in previous work [5, 6], the genomic sequences of which were downloaded from the NCBI SRA database, and the details are provided in Supplementary Data Table S2. The geographic distributions of apricot populations used in this study are labeled in Fig. 1A.

### Genome *de novo* assembly and annotation

First, we checked the quality of the raw data using Smrtlink software and removed poor-quality sections based on the signal-to-noise ratio (SNR). We selected reads longer than 1000 bp for assembly using hifiasm software with default settings [61]. To build a high-quality chromosome-level assembly of PHP, we aligned the Hi-C reads using juicer programs against the hifiasm-assembled contig-level genome [61, 62]. The 3D-DNA pipeline was used to correct misjoined contigs and construct scaffolds [63]. The reference-guided software RagTag was used to correct the assembly of the PZH genome with default parameters [19]. Finally, the two assemblies were reviewed using Juicebox Assembly Tools (JBAT) [64] with correction by manual modification. BUSCO software was used to assess the level of genome completeness of PHP and PZH [65]. For gene annotation, we integrated three methodologies: homology-based annotation utilizing five homologous species (*Arabidopsis thaliana*, *Oryza sativa*, *Populus trichocarpa*, *Malus* × *domestica*, *P. armeniaca* Stella and Marouch); transcriptome-based analysis; and *ab initio* predictions following the method of Huang *et al*. [66].

### Genome evolutionary analyses

Chromosomes from PHP and PZH assemblies were separately aligned to the *P. armeniaca* genome using minimap2 software [67]. Structural rearrangements were identified using SyRI with standard parameters and visualized with plotsr [20, 68]. The synonymous substitution rates (*K*_s_) between pairs of collinear genes, which were identified by using MCSCANX with BLASTp e-value 1e−5 [69], were computed using KaKs_Calculator 2.0 following the YN model [70]. The orthologous groups among 12 species (*A. thaliana*, *P. trichocarpa*, *Vitis vinifera*, *Rosa chinensis*, *Fragaria vesca*, *P. persica*, *P. mume*, *P. mandshurica*, *P. sibirica*, *P. armeniaca*, and our new assembly genomes of PHP and PZH) were identified using OrthoFinder with default parameters [71]. The identified single-copy orthologs were aligned by MAFFT and we constructed the maximum likelihood phylogenetic tree using IQ-TREE [72, 73]. Divergence timing among species was estimated using MCMCtree [74]. Three fossil calibration points were selected according to a previous study [43]. Furthermore, we identified gene family expansion and contraction using CAFE5 with default parameters [75].

### Variant detection and population genetic and phylogenetic analysis

For the newly sequenced and NCBI download reads, we used Trimmomatic to remove adapters and cut off bases, and discard reads shorter than 36 bases [76]. Approximately 300 Gb of high-quality reads were retained for the subsequent analysis. The clean reads were aligned to the latest high-quality apricot genome [5]. The Sentieon DNAseq pipeline was used to conducted the raw SNP, and then the raw SNP was filtered using VCFtools with parameters ‘--max-missing 0.2 --mac 3 --minQ 20’ [77, 78], and a final population dataset with 18 187 408 SNPs was obtained. PCA was performed using PLINK [79]. Nucleotide diversity (*π*), population fixation statistics (*F*_ST_), and Tajima’s *D* statistics were computed utilizing VCFtools [77]. We utilized PopLDdecay to compute the decay of LD, relying on allele frequency correlations (*r*^2^) [80]. The analysis of population genetic structure was conducted employing ADMIXTURE [81], considering a range of *K* values spanning from 2 to 10. A hypothetical natural hybrid individual of PHP was found in the structure analysis and was excluded from the subsequent analysis. The VCF file was transformed into a PHYLIP matrix employing the vcf2phylip.py script [82]. The phylogenetic tree was constructed using IQ-TREE with an ascertainment bias correction (+ASC) model [73, 83].

### Population divergence and demographic history

Population divergence times were estimated using the SNAPP package in BEAST 2 [84, 85]. Two chains of SNAPP analysis were run with 500 000 generations, sampled every 50 generations, and calibrated with a lognormal distribution centered at 4.19 Ma in the crown [5]. The population demographic history of each population was calculated based on the site-frequency spectrum (SFS), which was generated using ANGSD software [86]. In addition, Stairway Plot 2 was used to infer the historical effective population size (*N*_e_) with 200 bootstraps, a generation time of 5 years, and a mutation rate of 4.46e−9 as previously estimated [5, 21].

### Heterozygosity, inbreeding level, and deleterious variants

We estimated the expected heterozygosity (*H*_e_) and inbreeding coefficient (*F*_IS_) for each individual using VCFtools [77]. Protein-coding variants were annotated and classified into three categories using the SnpEff: LOF, missense, and synonymous variants [87]. We used the SIFT 4G program to predict harmful missense mutations [25]. Mutations with a SIFT score <0.05 were seen as harmful, while those with a score >0.05 were considered tolerated. We then calculated the mean the frequencies of harmful and less harmful mutations in each individual.

### Gene flow analysis

To estimate the population migration events, TreeMix analysis was performed with values of ‘-m’ ranging from 1 to 5 [88]. We also produced ABBA-BABA-related statistics using *P. mume* as the outgroup, implemented in Dsuite software with the ‘Dtrios’ function [89]. To investigate different models of gene flow between PHP/PZH and the NW_CA apricot clade, we examined seven alternative 2D models (visualized in Supplementary Data Fig. S5) using dadi with default parameters [90, 91]. To delineate distinct genomic regions potentially experiencing introgression between PHP and the NW_CA clade, the Python script ABBABABAwindows.py was employed to compute modified *f*_d_ statistics using a grid size of 100 kb [92]. Following previous studies [92, 93], results with a negative Patterson’s *D* statistic and *f*_d_ > 1 were ignored. The top 5% of windows were considered as the genomic regions with significant signals of introgression between PHP and the NW_CA apricot clade, which were further used for Gene Ontology (GO) enrichment analysis.

### Environmental differentiation, selective sweep and Gene Ontology enrichment analysis

To predict the environmental differentiation of PHP/PZH from the other apricot populations based on various climate variables, the occurrence records of CA_N_Par, CA_S_Par, NW_Psib, and NE_Psib were obtained as reported in previous research [17]. Records for mei were downloaded via Zhang *et al*. [6]. The occurrence records of PHP and PZH were gathered from our field research. In total, eight climatic variables and elevation information were downloaded from WorldClim and we quantified the environmental differentiation through PCA performed in R. To detect signs of selection within the genomic regions of PHP and PZH, we employed a CLR test utilizing SweeD with a grid size of 2 kb [94]. The top 5% of values, constituting the maximum CLR score, were employed as the CLR threshold for the identification of sweep regions. These candidate genes were subsequently corroborated using Tajima’s *D* statistic, *F*_ST_ analysis, and cross-population extended haplotype homozygosity (XP-EHH) [95]. Tajima’s *D* statistic and *F*_ST_ analysis were calculated by VCFtools, and the XP-EHH test was performed in selscan software [96]. GO and KEGG enrichment analysis of genomic regions with signatures of selection was implemented in the R package clusterProfiler [97].

## Supplementary Material

Web_Material_uhad215
