# Supplementary material for: Genomic evidence for evolutionary history and local adaptation of two endemic apricots: Prunus hongpingensis and P. zhengheensis
Source: Hortic Res. 2023 Oct 27;11(4):uhad215. doi: 10.1093/hr/uhad215 (PMC11059793; doi:10.1093/hr/uhad215)
Supplement: Web_Material_uhad215 [file web_material_uhad215.zip › Fig.S2.pdf]

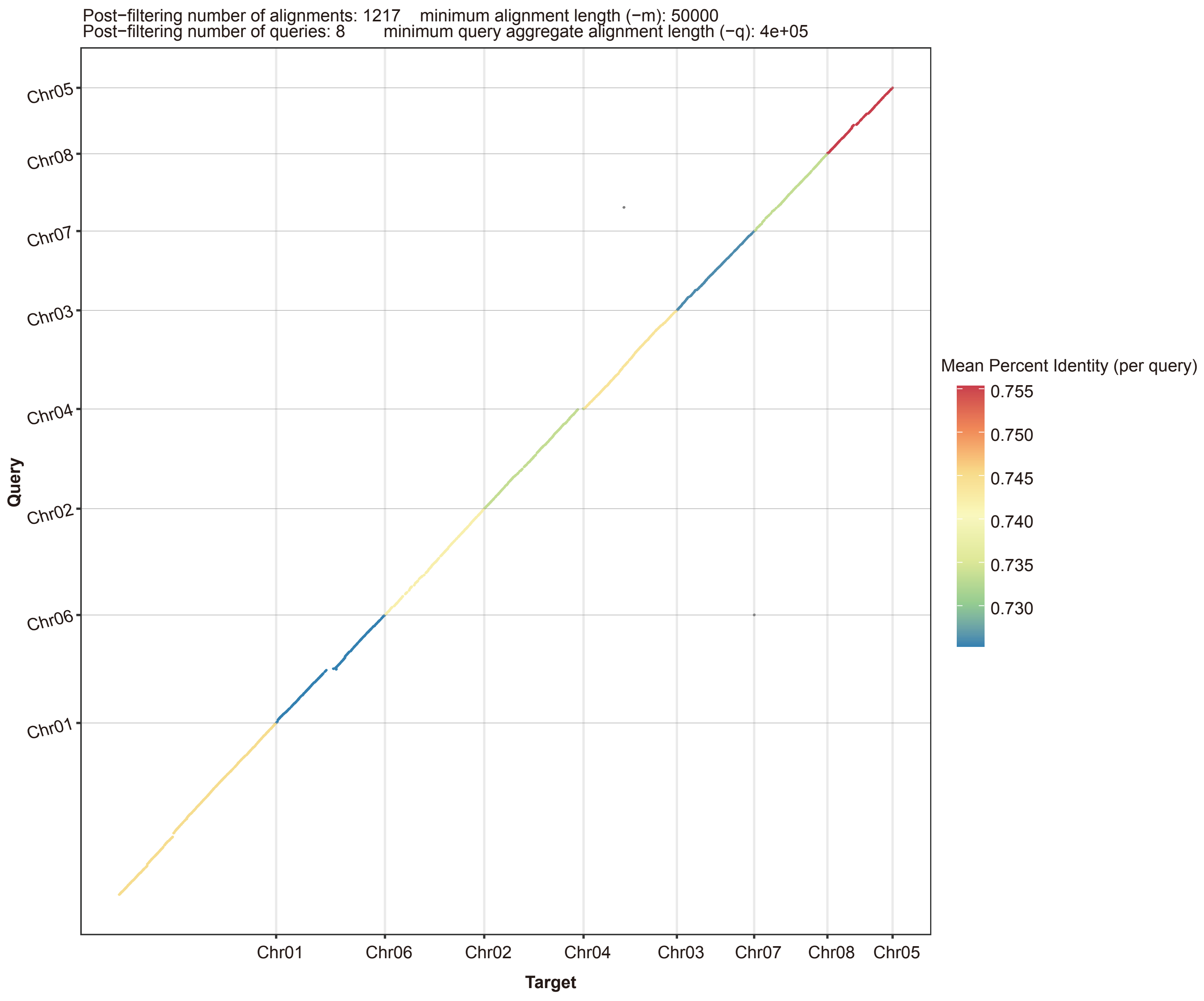

Post−filtering number of alignments: 1217 minimum alignment length (−m): 50000
Post−filtering number of queries: 8 minimum query aggregate alignment length (−q): 4e+05
Chr05
Chr08
Chr07
Chr03
Chr04
Chr02
Chr06
Chr01
Query
Chr01
Chr06
Chr02
Chr04
Chr03
Chr07
Chr08
Chr05
Target
Mean Percent Identity (per query)
0.755
0.750
0.745
0.740
0.735
0.730
