# Supplementary material for: Genomic evidence for evolutionary history and local adaptation of two endemic apricots: Prunus hongpingensis and P. zhengheensis
Source: Hortic Res. 2023 Oct 27;11(4):uhad215. doi: 10.1093/hr/uhad215 (PMC11059793; doi:10.1093/hr/uhad215)
Supplement: Web_Material_uhad215 [file web_material_uhad215.zip › Fig.S5.pdf]

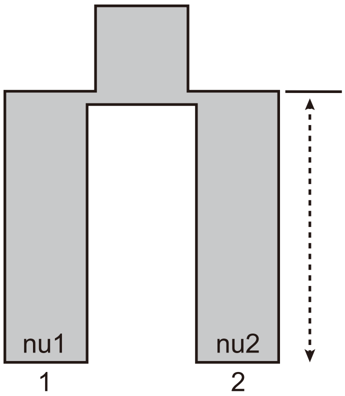

Divergence with no migration
“no_mig”

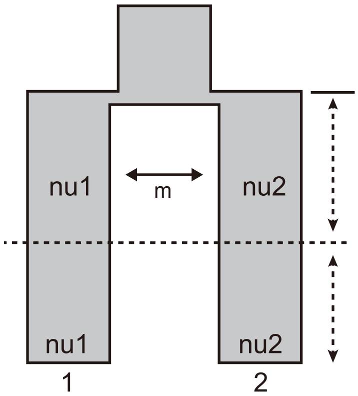

Divergence with ancient continuous symmetrical migration, isolation
“anc_sym_mig”

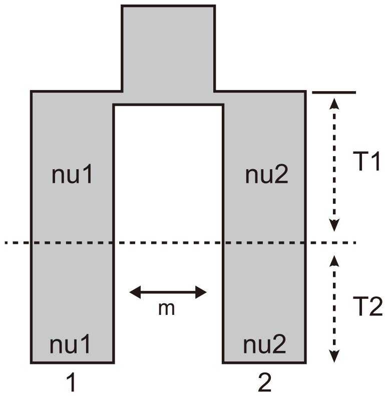

Divergence in isolation, continuous symmetrical secondary contact
“sec_contact_sym_mig”

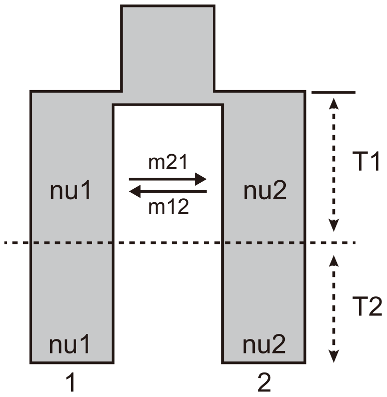

Divergence with ancient continuous asymmetrical migration, isolation
“anc_asym_mig”

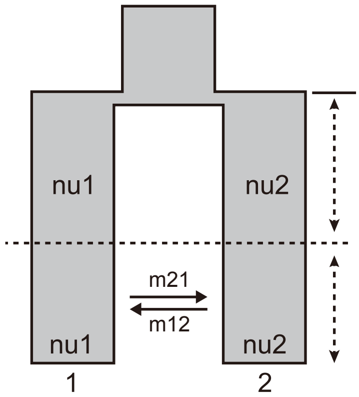

Divergence in isolation, continuous asymmetrical secondary contact
“sec_contact_asym_mig”

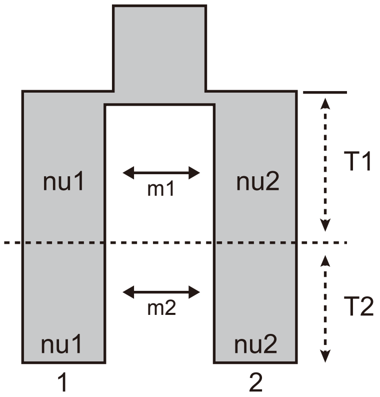

Divergence with continuous symmetric migration that varies across two epochs
“sym_mig_twoepoch”

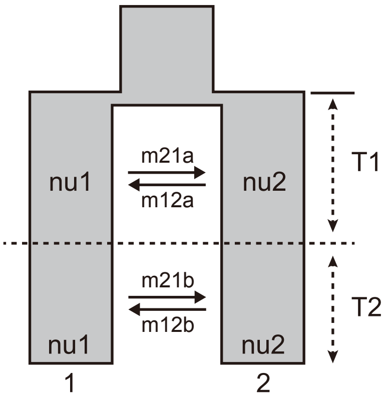

Divergence with continuous asymmetric migration that varies across two epochs
“asym_mig_twoepoch”
