# Supplementary figures and images for: Genomic evidence for evolutionary history and local adaptation of two endemic apricots: Prunus hongpingensis and P. zhengheensis
Source: Hortic Res. 2023 Oct 27;11(4):uhad215. doi: 10.1093/hr/uhad215 (PMC11059793; doi:10.1093/hr/uhad215)

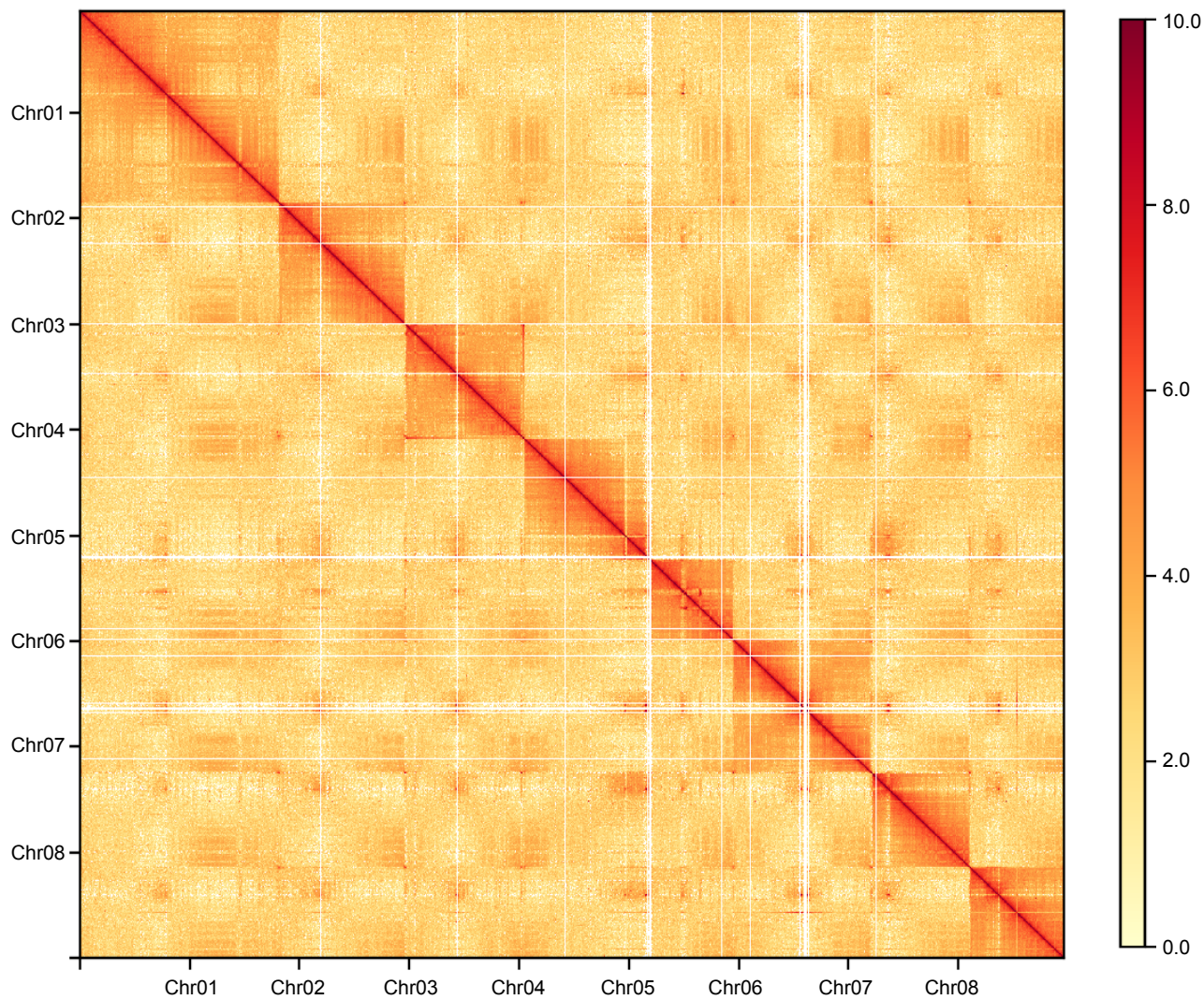

Supplement: Web_Material_uhad215 [file web_material_uhad215.zip › Fig.S1.pdf]

**A**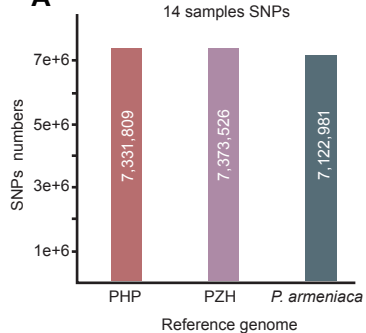**B**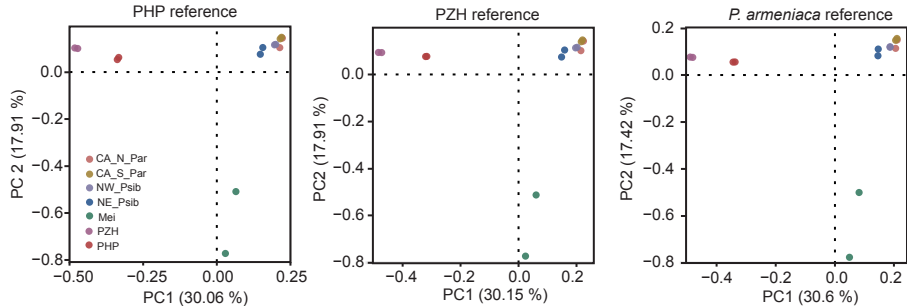

Supplement: Web_Material_uhad215 [file web_material_uhad215.zip › Fig.S3.pdf]

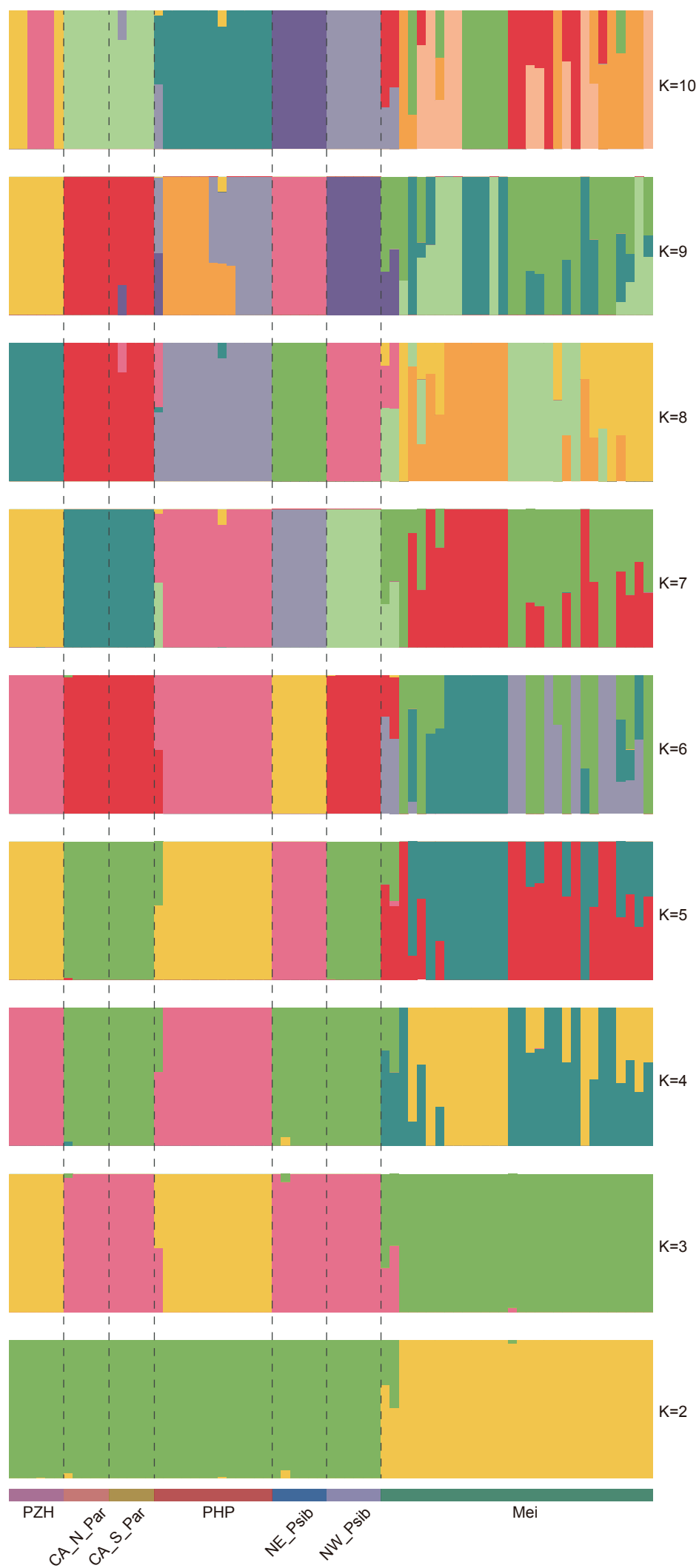

Supplement: Web_Material_uhad215 [file web_material_uhad215.zip › Fig.S4.pdf]

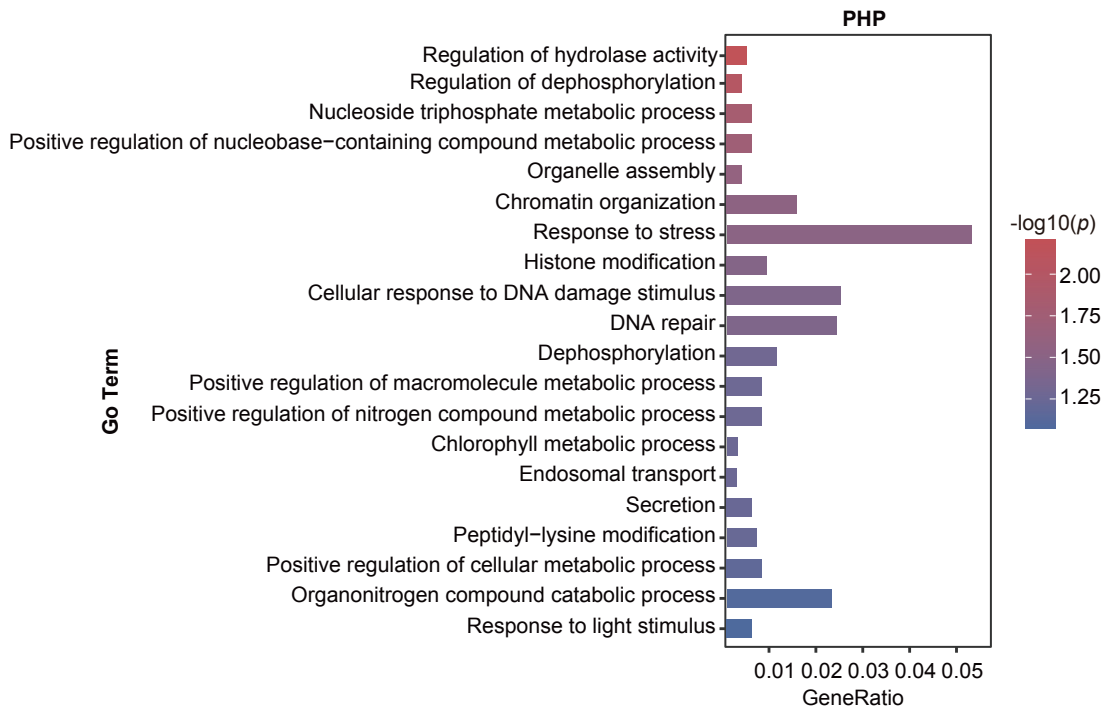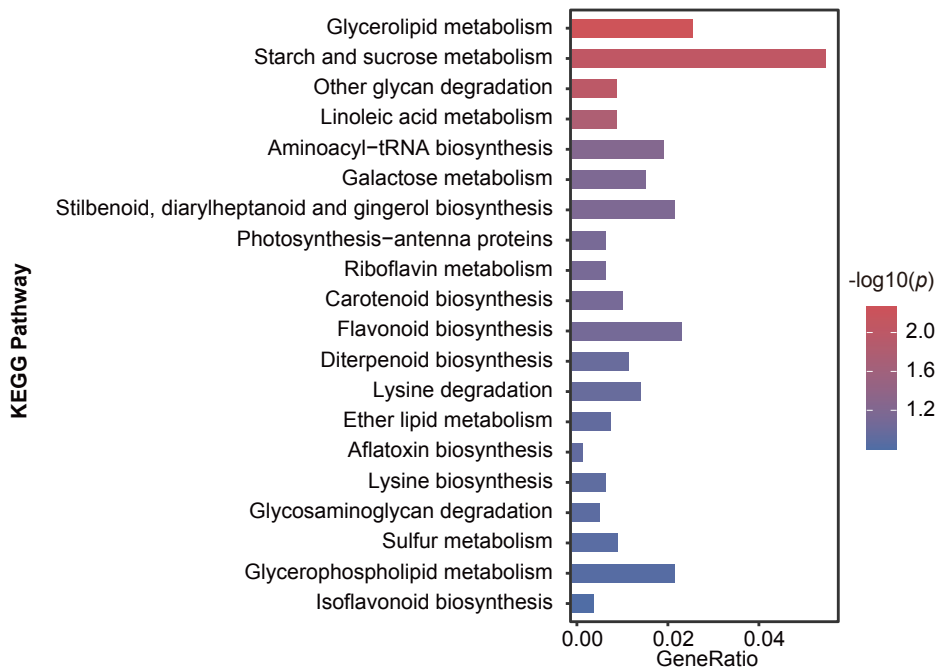

Supplement: Web_Material_uhad215 [file web_material_uhad215.zip › Fig.S6.pdf]

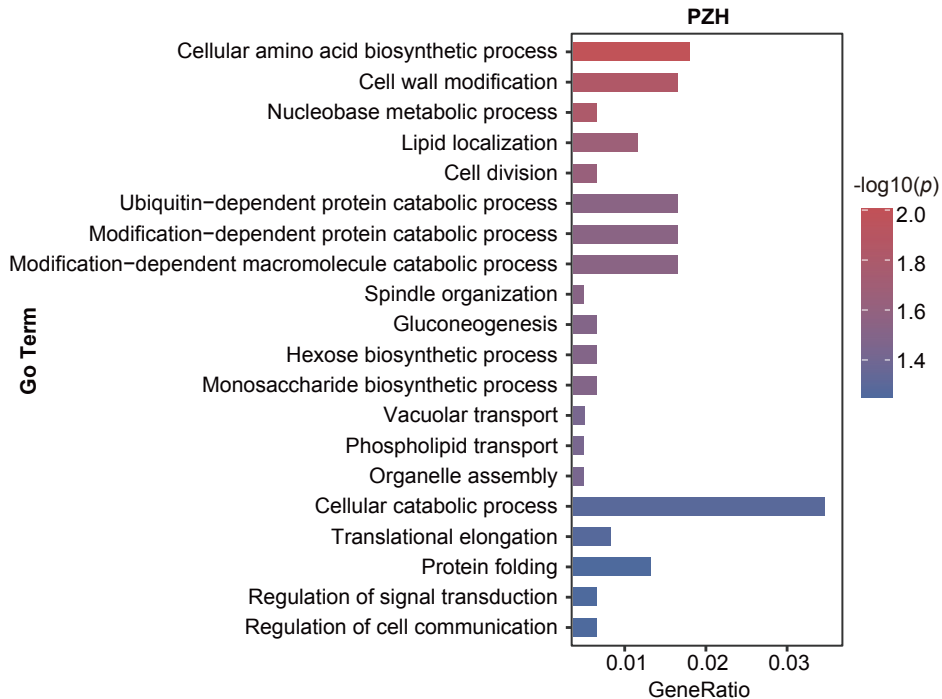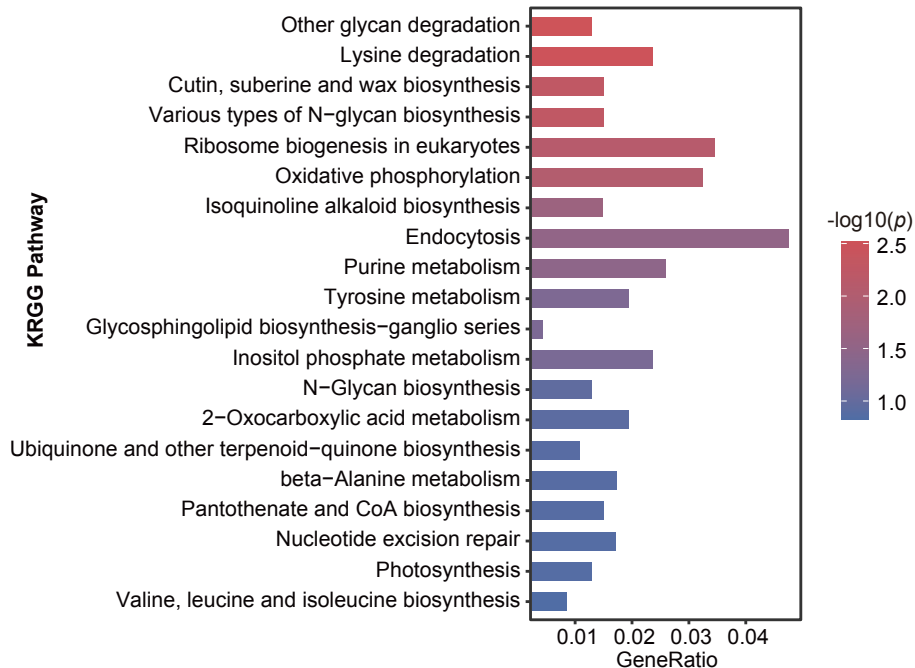

Supplement: Web_Material_uhad215 [file web_material_uhad215.zip › Fig.S7.pdf]
